# Demographic characteristics and spatial clusters of recent HIV-1 infections among newly diagnosed HIV-1 cases in Yunnan, China, 2015

**DOI:** 10.1186/s12889-019-7557-8

**Published:** 2019-11-11

**Authors:** Min Chen, Yanling Ma, Huichao Chen, Jie Dai, Hongbing Luo, Chaojun Yang, Lijuan Dong, Xiaomei Jin, Min Yang, Li Yang, Lijun Song, Manhong Jia, Zhizhong Song

**Affiliations:** Institute for AIDS/STD Control and Prevention, Yunnan Center for Disease Control and Prevention, No 158, Dongsi Street, Xishan District, Kunming, 650022 Yunnan China

**Keywords:** Human immunodeficiency virus (HIV), Recent infection, Spatial cluster, Yunnan

## Abstract

**Background:**

The characteristics of recent HIV infections can provide the information about the dynamics of HIV transmission. Yunnan is one of the provinces hardest-hit by HIV-1 in China. To further understand the characteristics of the HIV-1 epidemic in Yunnan, we analyzed the prevalence of recent HIV-1 infections among newly diagnosed cases, identified the associated factors and explored the spatial distribution of recent HIV-1 infections.

**Methods:**

Residual plasma samples from HIV-1 diagnostic tests were preserved. The associated information was collected from China HIV/AIDS case reporting system. Recent HIV-1 infections were estimated by combining the information about disease progression and BED- capture enzyme immunoassay (CEIA). The proportions of recent HIV-1 infections among newly diagnosed cases stratified by demographic characteristics were analyzed. The spatial clusters of recent HIV-1 infections were investigated by spatial scan statistics.

**Results:**

Among 6119 HIV/AIDS cases were newly reported between January 2015 and June 2015 in Yunnan Province, 9.3% (570/6119) were estimated as recent infections. Female, aged below 25 years and homosexual contact were more associated with the higher proportion of recent HIV-1 infections. Among the different demographic sub-groups, men who have sex with men (MSM) aged < 25 years and ≥ 50 years had a higher chance of being diagnosed as recent infections, heterosexually infected men aged ≥25 years had a lower chance of being diagnosed as recent infections. In the sub-groups with different screening approaches, the highest proportion of recent infections (16.1%) was found among women diagnosed by testing during pregnancy and childbirth. In the sub-groups with different contact histories, the higher proportion of recent infections was found among the female cases having commercial heterosexual contacts (16.4%) and MSM (19.7%). The statistically significant spatial clusters of recent infections attributed to heterosexual contact, homosexual contact and intravenous drug injection were identified, respectively.

**Conclusions:**

The investigation of recent HIV infections among newly diagnosed cases supplements the routine HIV surveillance, and reveals the characteristics of ongoing HIV transmission. Our finding identified the potential sub-populations and geographic areas in need of services or improved interventions.

**Electronic supplementary material:**

The online version of this article (10.1186/s12889-019-7557-8) contains supplementary material, which is available to authorized users.

## Background

Yunnan, a southwest province in China, has been most hit by Human Immunodeficiency Virus 1 (HIV-1) since the first HIV-1 epidemic was found among local intravenous drug users (IDUs) in 1989 [1–3]. After 2006, unprotected sexual contact replaced intravenous injection and became the main HIV transmission route in Yunnan [2]. By the end of 2014, the number of annually reported HIV/AIDS cases in Yunnan (11,225) dropped to second place, but the number of people living with HIV/AIDS in Yunnan (80,610) kept the first place in China. Yunnan has a population of about 48.0 million, HIV screening was scaled up from 4.835 million person-times in 2011 to 9.943 million person-times in 2015. The estimated proportion of discovered infected individuals increased from 68.6% in 2011 to 72.9% in 2015. Under the background of the 90–90-90 target for 2020, there is still a big gap to achieve the first 90 in Yunnan.

HIV prevalence and incidence are essential to evaluate HIV epidemic. Prevalence refers to the number of persons living with HIV at a given time. Incidence measures the number of new infections during a specified time, which reflects the extent of ongoing HIV transmission in a population. Among the approaches to estimate HIV incidence, the laboratory-based HIV incidence estimation involves a laboratory test for recent HIV infection combined with a mathematical formula to derive HIV incidence [4, 5]. BED-capture enzyme immunoassay (BED-CEIA) is a commonly used laboratory test for recent HIV-1 infection, which is based on measurement of the proportion of HIV-1-specific IgG to total IgG after seroconversion [6].

In China, newly diagnosed HIV/AIDS cases are required to be reported into the web-based HIV/AIDS case reporting system, whose purpose is to record demographic and health data of reported HIV/AIDS cases and follow-up information. The case reporting system was instituted in 2005, and integrated into China’s HIV/AIDS Comprehensive Response Information Management System (CRIMS) which launched in 2008 [7]. To improve the data quality, local-level public health workers are acquired to be trained with normative data reporting. China Center for Disease Control and Prevention organizes the assessment of data quality each year. The assessment is sequentially carried out by CDC staff at the county, prefecture and province levels. Because the case reporting system cannot provide the exact number of persons receiving HIV screening, HIV incidence estimation based on recent HIV infection is not available. However, recent HIV infections discriminating from newly reported cases also provide the information about HIV transmission dynamics over a recent period, and can supplement the method of estimating the HIV epidemic. The factors associated with recent infections can provide the clues for accurate HIV testing and intervention. To reach the first 90, 90% of people who are HIV infected will be diagnosed, it is necessary to improve the efficiency in HIV detecting by find out the key sub-populations. Thus, in this study, the demographic characteristics and spatial distribution of recent HIV infections among HIV cases newly diagnosed in Yunnan between January and June 2015 were investigated for the first time.

## Methods

### Study participants and sample collection

The study process followed the previous study [8]. Briefly, a total of 6119 plasma samples of newly reported HIV/AIDS cases were collected in Yunnan between January 2015 and June 2015. Among them, 4540 samples whose CD_4_^+^ T lymphocytes counts were more than 200 cells/μl were further tested for the recent infection status. The written informed consent was obtained. The study was approved by the Biomedical Ethics Review Committee of Yunnan Province.

### Recent HIV-1 infection identified with BED-capture enzyme immunoassay (CEIA)

The procedure of BED-CEIA (Calypte HIV-1 BED incidence EIA, Calypte Biomedical Corporation, Portland, OR) was previously described [8]. After the screening and confirmatory testing, the specimens were judged as recent infections within 168 days after seroconversion or long-term infections beyond 168 days after seroconversion.

### Spatial scan statistics analysis for HIV-1 recent infections

The spatial scan statistics analysis for HIV-1 recent infections was performed by SaTScan [9], which can detect potential spatial clusters by automatically scanning study area with a continually varying window. Among the windows with statistical significance which was evaluated using Monte Carlo simulation, the window with the maximum likelihood is the most likely cluster (primary cluster), and other windows with smaller likelihood are secondary clusters. In this study, HIV-1 cases’ addresses were based on current living place. According to the previous studies, a Poisson-based model was chosen [10–14]. The circular scanning window was usually used [10, 12, 15, 16]. As an alternative to the circle, the ellipse scanning window provides slightly higher power for true clusters that are long and narrow in shape, and slightly lower power for circular and other very compact clusters [9]. We compared the results by using the two kinds of scanning windows. The significant clusters of homosexual contact and intravenous drug use were same when using the two kinds of scanning windows. As shown in Additional file 1, the primary cluster of heterosexual contact was neither affected by the choices of the scanning windows. Only one significant secondary cluster of heterosexual contact was found by using the ellipse scanning window. However, this secondary cluster was divided into two parts by one county which was not integrated in this cluster. This suggested that the circular scanning window better performs with our data. The maximum spatial cluster size widely used in the literatures is 50% of the population at risk [11–14, 16–18]. To decide the maximum spatial cluster size, a sensitivity analysis was performed by setting the maximum spatial cluster size as 5, 10, 25 and 50% of the population at risk in the spatial window, respectively. As shown in Additional file 2, when using 5% of the population at risk, the primary cluster of heterosexual contact was smaller than those when using 10, 25 and 50% of the population at risk. However, the significant clusters of heterosexual contact were no difference when using 10, 25 and 50% of the population at risk. The significant clusters of homosexual contact and intravenous drug use were same under the different window sizes. Therefore, 10% of the population at risk was chosen as the maximum spatial cluster size. The detected spatial clusters were shown on maps with Quantum GIS [19].

### Statistical analysis

Statistical analyses were conducted with the SPSS 21.0 statistical analysis software package (SPSS Inc. Chicago, IL). Univariate logistic analysis was first performed, based on which variables with *p* < 0.10 were selected for multivariate logistic analysis with a backward unconditional method. All tests were two-tailed and a *p* value < 0.05 was considered statistically significant.

## Results

### Demographic characteristic of study participants

A total of 6119 HIV/AIDS cases were newly reported in Yunnan Province between January 2015 and June 2015. By combining the information about disease progression and BED-CEIA assay, 570 samples were estimated as recent infections.

Among the 570 individuals, 60.5% (345/570) were males, and 39.5% (225/570) were females; the median age was 35 years (range: 15–84 years); Chinese accounted for 91.4% (521/570), foreigners accounted for 8.6% (49/570); and 58.8% (335/570) of individuals were Han ethnicity, while 41.2% (235/570) were minorities; Peasants accounted for 67.2% (383/570). Of them, 32.1% (183/570) were single, 46.7% (266/570) were married, and 21.2% (121/570) were divorced or widowed. Heterosexual contact was the major transmission route, accounting for 80.5% (459/570), while homosexual contact and intravenous drug injection accounted for 9.5% (54/570) and 9.6% (55/570), respectively.

### Demographic characteristic associated with recent HIV-1 infections

The overall proportion of recent infections among the newly diagnosed cases was 9.3% (570/6119). As shown in Table 1, the proportions of recent infections showed no statistical differences among newly diagnosed cases stratified by nationality, ethnicity, occupation and marital status. The proportion of recent infections among women (11.2%, 225/2003) was significantly higher than that among men (8.4%, 345/4116). Within the different age classes, the highest proportion of recent infections was found among the cases aged below 25 years (16.7%, 117/700). Within the different transmission routes, the highest proportion of recent infections was found among the homosexually infected cases (19.7%, 54/274).

**Table 1:** Demographic characteristics of recent HIV infections among newly diagnosed cases between January and June 2015

	Total	Resent infections	The proportion of recent infecions	Univariate analysis		Multivatiate analysis	
				*p*	OR (95% CI)	*p*	OR (95% CI)
Gender							
Male	4116	345	8.4%	-	1.000	-	1.000
Female	2003	225	11.2%	<0.001	1.383 (1.159~1.651)	<0.001^a^	1.514 (1.253~1.830)
Age							
<25	700	117	16.7%	-	1.000	-	1.000
25-49	3966	338	8.5%	<0.001	0.464 (0.370~0.583)	<0.001^a^	0.553 (0.436~0.702)
≥50	1453	115	7.9%	<0.001	0.428 (0.325~0.564)	<0.001^a^	0.550 (0.411~0.736)
Nationality							
Chinese	5613	521	9.3%	-	1.000		
Foreigners	506	49	9.7%	0.766	1.048 (0.770~1.426)		
Race/ethnicity							
Han	3692	335	9.1%	-	1.000		
Others	2427	235	9.7%	0.423	1.074 (0.902~1.280)		
Ocuppation							
Peasants	4138	383	9.3%	-	1.000		
Others	1981	187	9.4%	0.817	1.022 (0.850~1.228)		
Marital Status							
Unmarried	1618	183	11.3%	-	1.000		
Married	3264	266	8.1%	<0.001	0.696 (0.570~0.848)		
Divorced/Widowed	1235	121	9.8%	0.195	0.852 (0.668~1.086)		
Unknown	2	0	0.0%	0.999	-		
Infection Routes							
Heterosexual contact	5297	459	8.7%	-	1.000	-	1.000
Homosexual contact	274	54	19.7%	<0.001	2.587 (1.892~3.537)	<0.001^a^	2.454 (1.739~3.461)
Intravenous drug injection	525	55	10.5%	0.164	1.233 (0.918~1.657)	0.058	1.345 (0.990~1.827)
Unknown	23	2	8.7%	0.996	1.004 (0.235~4.295)	0.922	1.076 (0.250~4.627)

^a^statistical significance

Based on the combinations of transmission routes, gender and age, the newly reported cases were divided into different sub-groups, as shown in Table 2. A logistic regression was performed to evaluate the proportions of recent HIV-1 infections in the different sub-groups. When compared with heterosexually infected men aged below 25 years, MSM aged below 25 years and aged above 49 years had a higher chance of being diagnosed as recent infections, heterosexually infected men aged 25 years and above had a lower chance of being diagnosed as recent infections, the rest sub-groups showed no statistical differences.

**Table 2:** The proportion of recent HIV infections among the different sub-groups of newly diagnosed cases

Sub-groups			Total	Recent Infections	The proportion of recent infections	*p*	OR (95% CI)
Heterosexual contact	Men	<25 years	208	26	12.5%	-	1.000
		25-49 years	2155	144	6.7%	0.002^a^	0.501 (0.321~0.782)
		≥50 years	993	71	7.2%	0.011^a^	0.539 (0.335~0.868)
	Women	<25 years	290	49	16.9%	0.178	1.423 (0.852~2.377)
		25-49 years	1226	133	10.8%	0.483	0.852 (0.544~1.334)
		≥50 years	425	36	8.5%	0.111	0.648 (0.380~1.105)
Homosexual contact	Men	<25 years	124	32	25.8%	0.002^a^	2.435 (1.370~4.327)
		25-49 years	143	18	12.6%	0.981	1.008 (0.530~1.917)
		≥50 years	7	4	57.1%	0.005^a^	9.333 (1.976~44.076)
Intravenous drug use	Men	<25 years	68	8	11.8%	0.873	0.933 (0.401~2.172)
		25-49 years	373	37	9.9%	0.339	0.771 (0.452~1.314)
		≥50 years	26	3	11.5%	0.888	0.913 (0.256~3.256)
	Women	<25 years	7	2	28.6%	0.233	2.800 (0.516~15.183)
		≥25 years	51	5	9.8%	0.596	0.761 (0.277~2.090)
Others	-	-	23	2	8.7%	0.598	0.667 (0.148~3.010)

^a^statistical significance

### Proportions of recent HIV-1 infections in sub-groups with different screening approaches

Combined with gender, the sources of reported HIV-1 cases were divided into nine categories, including voluntary counseling and testing (VCT) for men and women, provider-initiated HIV testing and counseling (PITC) for men and women, pre-marital medical checking for men and women, testing for women during pregnancy and childbirth, testing for men and women with HIV-1 positive spouses or sexual partners and other testing for men and women. As shown in Table 3, the proportion of recent HIV-1 infections among the male cases discovered by PITC (6.7%) was the lowest, and that among the female cases discovered by testing during pregnancy and childbirth (16.1%) was the highest.

**Table 3:** The proportion of recent HIV infections from the different screening approaches

Sample sources		Total	Recent Infections	The proportion of recent infections	*p*	OR (95% CI)
Men	Voluntary counseling and testing (VCT)	779	79	10.1%	-	1.000
	Provider-initiated HIV Testing Counseling (PITC)	2068	139	6.7%	0.002^a^	0.638 (0.478~0.853)
	Pre-marital medicalchecks	244	22	9.0%	0.607	0.878 (0.535~1.442)
	Testing for spouses or sexual partners of HIV-positive persons	129	11	8.5%	0.570	0.826 (0.427~1.598)
	Others	896	94	10.5%	0.814	1.039 (0.757~1.424)
Women	Voluntary counseling and testing (VCT)	424	50	11.8%	0.377	1.185 (0.814~1.725)
	Provider-initiated HIV Testing Counseling (PITC)	860	80	9.3%	0.567	0.909 (0.655~1.261)
	Pre-marital medicalchecks	113	13	11.5%	0.656	1.152 (0.618~2.148)
	Testing during pregnancy and childbirth	211	34	16.1%	0.016^a^	1.702 (1.102~2.628)
	Testing for spouses or sexual partners of HIV-positive persons	134	16	11.9%	0.529	1.201 (0.678~2.128)
	Others	261	32	12.3%	0.338	1.238 (0.800~1.917)

^a^statistical significance

### Proportions of recent HIV-1 infections in sub-groups with different contact histories

For women, the contact histories included having HIV-1 positive spouse/fixed sexual partner, non-commercial and non-marital heterosexual contact, commercial heterosexual contact, intravenous drug use and the others. For men, besides the above five, the other contact history was homosexual contact. As shown in Table 4, the proportions of recent HIV-1 infections among the male cases who had homosexual contacts (19.7%) and among the female cases who had commercial heterosexual contacts (16.4%) were higher than the other nine subgroups.

**Table 4:** The proportion of recent HIV infections in the sub-groups with different contact histories

Contact history		Total	Recent Infections	The proportion of recent infections	*p*	OR (95% CI)
Men	Having HIV positive spouse/fixed sexual partner	208	17	8.2%	-	1.000
	Non-commercial and non-marital heterosexual contact	1931	139	7.2%	0.608	0.871 (0.515, 1.474)
	Commercial heterosexual contact (Clients)	1217	85	7.0%	0.539	0.844 (0.490, 1.452)
	Having sex with men	274	54	19.7%	0.001^a^	2.758 (1.546, 4.918)
	Introvenos drug use	467	48	10.3%	0.393	1.287 (0.721, 2.297)
	Others	19	2	10.5%	0.724	1.322 (0.281, 6.208)
Women	Having HIV positive spouse/fixed sexual partner	425	51	12.0%	0.147	1.532 (0.861, 2.725)
	Non-commercial and non-marital heterosexual contact	1370	143	10.4%	0.315	1.309 (0.774, 2.214)
	Commercial heterosexual contact (FSWs)	146	24	16.4%	0.019^a^	2.210 (1.141, 4.283)
	Introvenos drug use	57	7	12.3%	0.342	1.573 (0.618, 4.001)
	Others	5	0	0.0%	0.999	-

^a^statistical significance

### Spatial analysis of recent HIV-1 infections

**Fig. 1:**
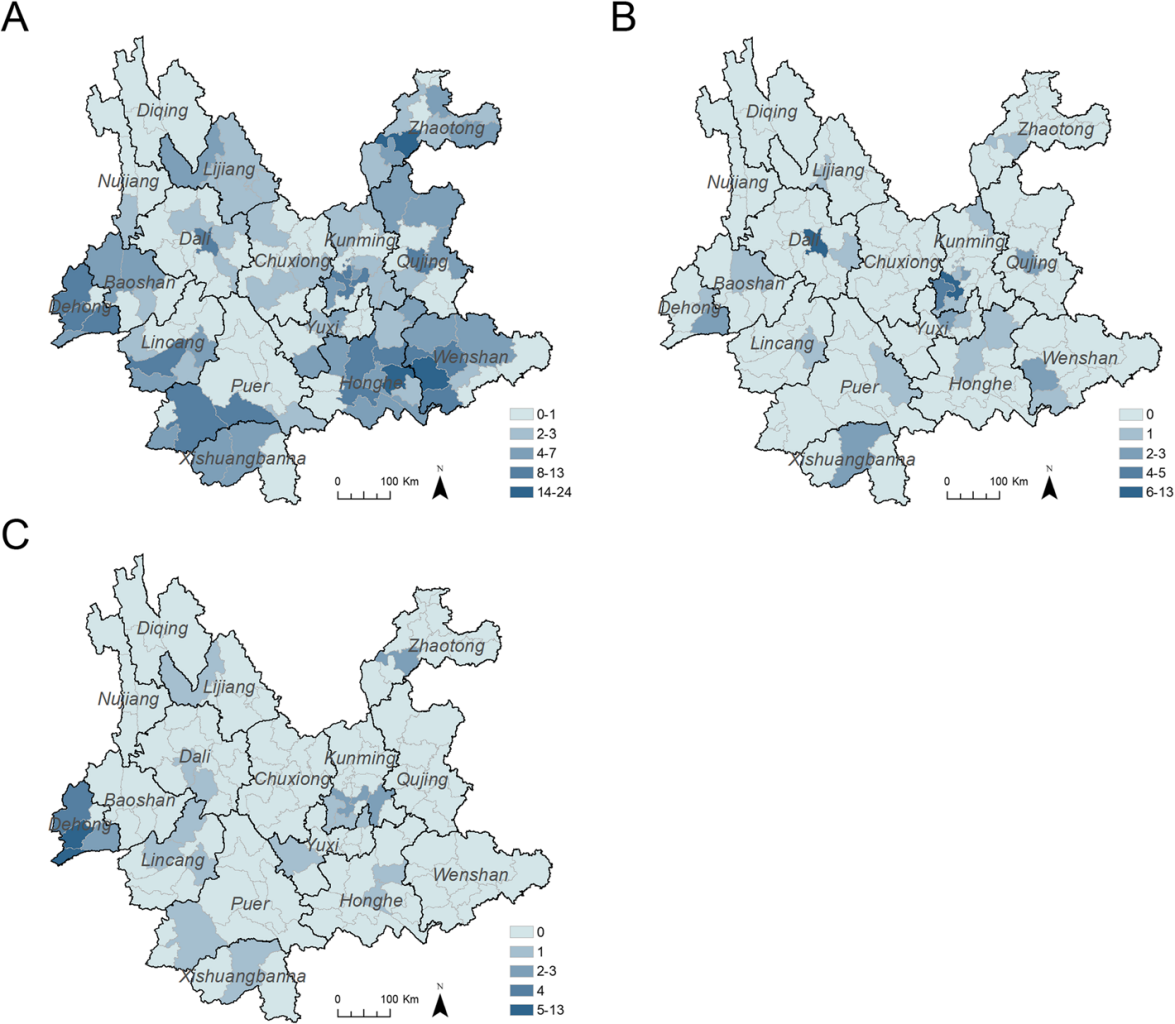
Choropleth maps of recent HIV infections attributed to different transmission routes at the county level between January and June 2015. **a** The choropleth maps of recent infections attributed to heterosexual contact at the county level. **b** The choropleth maps of recent infections attributed to homosexual contact at the county level. **c** The choropleth maps of recent infections attributed to intravenous drug use at the county level. The shapefile of China was downloaded from the GADM database (www.gadm.org), version 3.4, April 2018, from which the shapefile of Yunnan was extracted with Quantum GIS

To explore the spatial distribution of recent HIV-1 infections attributed to different infection routes, the estimated recently infected cases attributed to different infection routes were hierarchically mapped at the county level by Jenks natural breaks classification method, respectively. As shown in Fig. 1a, the recent infections attributed to heterosexual contact were lower in the center part of Yunnan, and higher in the southeast of Yunnan and the areas along the western border. As shown in Fig. 1b, the recent infections attributed to homosexual contact were higher in two neighboring counties in Kunming and one county in Dali Prefecture. However, the recent infections attributed to intravenous drug use were higher in four counties in Dehong.

**Fig. 2:**
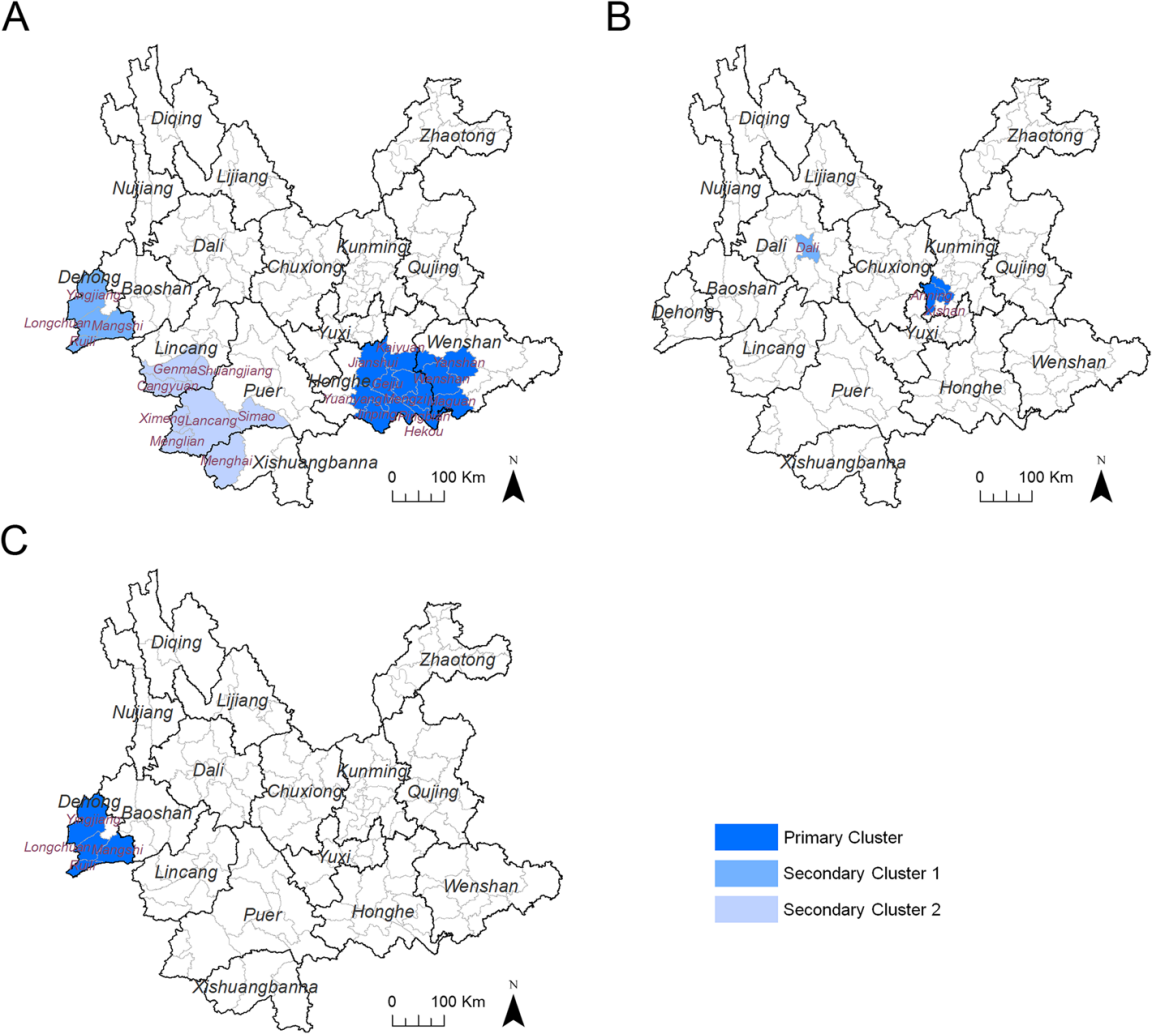
Spatial clusters of recent HIV infections attributed to the main transmission routes between January and June 2015. **a** The primary and secondary clusters of recent infections attributed by heterosexual contact. **b** The primary and secondary clusters of recent infections attributed by homosexual contact. **c** The primary clusters of recent infections attributed by intravenous drug use. The shapefile of China was downloaded from the GADM database (www.gadm.org), version 3.4, April 2018, from which the shapefile of Yunnan was extracted with Quantum GIS

To further discloses the spatial characteristics of the risks related to HIV-1 transmission, spatial clusters of recent HIV-1 infections attributed to the main infection routes were analyzed by using spatial scan statistics. Among recent infections attributed to heterosexual contact, three non-overlapping statistically significant clusters were detected: the most likely cluster (relative risk (RR) =3.17) included 11 counties in Honghe Prefecture and Wenshan Prefecture bordering Vietnam; the first and second secondary clusters (RR = 3.51 and 2.27) included four counties in Dehong Prefecture and eight counties in the southwest of Yunnan, respectively (Fig. 2a and Table 5). Among recent infections attributed to homosexual contact, two non-overlapping statistically significant clusters were detected: the most likely cluster (RR = 20.77) included two counties in Kunming Prefecture; the first secondary clusters (RR = 13.93) located in the capital city of Dali Prefecture (Fig. 2b and Table 5). For recent infections attributed to intravenous drug use, the only one most likely cluster (RR = 51.07) located in Dehong Prefecture (Fig. 2c and Table 5).

**Table 5:** The characteristics of spatial clusters of recent HIV infections attributed by the main transmission routes

Spatial Clusters	Counties	Number of cases	Relative Risk	Log Likelihood Ratio	*p*-value
Heterosexual contact					
Primary Cluster	Mengzi, Pingbian, Gejiu, Kaiyuan, Wenshan, Hekou, Yuanyang, Jinping, Yanshan, Jianshui, Maguan	108	3.17	44.1	< 1.0E-17
Secondary Cluster 1	Ruili, Longchuan, Mangshi, Yingjiang	35	3.51	18.2	1.4E-06
Secondary Cluster 2	Menglian, Ximeng, Lancang, Menghai, Cangyuan, Shuangjiang, Gengma, Simao	43	2.27	10.5	2.0E-03
Homosexual Contact					
Secondary Cluster 1	Anning, Xishan	18	20.77	34.0	7.7E-13
Secondary Cluster 1	Dali	9	13.93	14.6	4.3E-05
Intravenous drug injection					
Secondary Cluster 1	Ruili, Longchuan, Mangshi, Yingjiang	30	51.07	75.9	< 1.0E-17

## Discussion

In this study, the characteristics of HIV-1 recent infections in Yunnan Province were investigated at a cross section. The proportions of recent infections among newly diagnosed HIV-1 cases showed statistical differences among the groups stratified by gender, age and transmission routes. The spatial distribution of recent HIV-1 infections described at the county level. In the main transmission routes, the spatial clusters of HIV-1 recent infections were found. To our knowledge, this is the first province-wide study of recent HIV-1 infections based on newly reported HIV-1 cases in China.

Over the study period, the overall proportion of recent infections among the newly diagnosed cases in Yunnan was 9.3%, which was much lower than those found in European countries [20–22]. A recent study showed that the proportion of recent HIV infections among newly diagnosed HIV cases in Germany was found as 30.4% between 2008 and 2014 [20]. In fact, the proportion of recent infections could be affected by the HIV incidence and the frequency of HIV testing. Thus, a low proportion of recent infections may be due to a delayed diagnosis.

As observed in other studies [20, 22], the overall proportion of recent infections was higher in the young cases aged below 25 years (16.7%), and decreased with age, which could be that the time from infection to diagnosis is shorter in younger persons. We also found that the proportion of recent infections among the female cases was a little higher than that among the male cases (11.2% vs 8.4%). We have no evidence that HIV-1 incidence in women was higher than that in men. According to the data from sentinel surveillance in Yunnan, even in female sex workers, the HIV-1 incidence (0.46, 95% CI 0.38–0.54%) showed no difference with that in male STD clinic attendees (0.47, 95% CI 0.39–0.55%) [23, 24]. Among pregnant women, HIV-1 incidence was 0.09% (95% CI 0.07–0.12%) [25]. One of the reasons for the high proportion of recent infections among women might be that women have more chances for HIV testing, such as the routine HIV detection during pregnancy and childbirth and the active HIV detection for FSWs. As found in this study, in the sub-groups divided by screening approaches, the proportion of recent infections in the sub-group of women diagnosed by testing during pregnancy and childbirth was the highest (16.1%); in the sub-groups with different contact histories, the proportion of recent infections in the subgroup of the female cases who had commercial heterosexual contacts (FSWs) was higher (16.4%), however, that in the sub-group of the male cases who had commercial heterosexual contacts (Clients) was much lower (7.0%). The other reason may be that women more actively seek medical service. Unlike women, men seemed not so actively to seek testing offers for HIV. As observed in this study, the proportion of recent infections in the subgroup of heterosexually infected men aged 25 years and above was the lowest, just 6.8% (215/3148). Meanwhile, the percentage of this subgroup accounted for 51.4% (3148/6119) of the newly reported cases over the study period. The long-standing HIV infections and unawareness of HIV infections in such a large subgroup could result into potential HIV transmission.

In fact, late diagnosis of HIV infection, defined as an initial CD4^+^ T lymphocyte count < 200 cells/μl, among newly identified HIV/AIDS cases in China is a challenge for HIV control and prevention, which is detrimental to infected persons and to the public health. From 2010 to 2014, the proportions of late diagnosis among annual newly identified HIV/AIDS cases in China were 41.8, 42.1, 38.1, 36.8 and 35.5% [26]. Especially, the proportions of late diagnosis appeared high in medical settings, namely among cases discovered through PITC. In this study, we also found the proportion of recent HIV-1 infections among cases discovered through PITC was low, especially among the male cases discovered through PITC. Presently, early detection and diagnosis of HIV infection is a strategy for HIV control and prevention [27]. In China, HIV testing services are widely available, however, only 68% of all individuals with HIV were aware of their serostatus in 2015 [28]. To achieve the target of 90–90-90 strategy, HIV screening should be further scaled up [29, 30]. Meanwhile, HIV testing strategies should be optimized for more timely diagnosis of hard-to-reach populations. The information of recent infections among newly identified cases can provide the clues about which sub-population should be more attended. From the public health perspective, more efforts should be taken to increase HIV awareness of the public. Testing based on perceived risk of HIV infection is likely more efficient than broader screening approaches. Thus, besides testing in health care settings, the multiple testing modes should be developed, such as self-testing and self-sampling [31].

Strikingly, in the different transmission routes, the highest proportion of recent infections (19.7%, 54/274) was identified in MSM. This could be due to a higher HIV incidence among these sub-populations. A meta-analysis found that the national HIV incidence among Chinese MSM was 5.0 per 100 person-years (95% CI: 4.1–5.8%), which was much higher than that of any other sub-population in China [32]. Based on the sentinel surveillance in Yunnan, the HIV-1 incidence in MSM (7.02, 95% CI: 5.72–8.32%) estimated with BED-CEIA remained stubbornly high during 2008–2011, which was much higher than those in the other sub-populations [33]. A prospective cohort study also showed HIV-1 incidence among Yunnan MSM was 3.5 (95% CI: 1.8–6.2) per 100 person-years [34]. The high HIV-1 incidence among MSM constitutes a challenge to efforts to control the HIV-1 pandemic. Nowadays, the intervention for MSM mainly depended on the peers from non-government organizers. Perhaps, the internet/mobile apps-based health information delivery and testing servers should be enhanced, which provides maximum protection for their privacy [35]. According to a multicenter cross-sectional survey in China, young MSM (aged < 25 years old) had a higher prevalence of recent HIV-1 infection [36]. In this study, we found that the proportions of recent infections in the subgroups of MSM aged < 25 years (25.8%) and aged ≥50 years (57.1%) were much higher than the other subgroups, which suggested that older MSM also need to be concerned besides young MSM. Perhaps, different intervention modes should be taken.

By using spatial scan statistics, we further detected the spatial clusters of recent infections in the three main transmission routes. There were three statistically significant spatial clusters for heterosexual contact transmission, among which the primary cluster located in Honghe and Wenshan, the two secondary spatial clusters located in the west of Yunnan. These three clusters covered 17.8% (23/129) of counties in Yunnan, and included 40.5% (186/459) of recently infected cases attributed to heterosexual contact, which suggested that the intervention for heterosexual contact transmission should be further improved in these areas. As mentioned, heterosexual contact became the main transmission route in Yunnan after 2006 [1, 2]. The same change also saw in the whole nation, where HIV has been mainly circulating in the general population and heterosexual contact is the main drivers of HIV epidemic [3, 37]. Because of a large population having heterosexual behaviors, the dispersion of infection sources and the changes of social ideas, the contribution of new infections caused by heterosexual transmission to the total number of new infections in China is about to be significant in the years to come [38]. Interventions for heterosexual transmission need be further optimized and improved.

For homosexual contact transmission, two statistically significant spatial clusters were detected in three counties of Kunming and Dali, and included 50.0% (27/54) of recently infected cases attributed to homosexual contact. Furthermore, the RR of these clusters (20.77 and 13.93) were far higher than those of the clusters for heterosexual contact. RR is defined as a multiple of the estimated risk within the cluster over that outside the cluster [9]. For the two spatial clusters of homosexual contact, the estimated risks of recent HIV-1 infections caused by homosexual contact in clusters were 20.77 and 13.93 times as high as those outside clusters. This suggested that the risk of homosexual transmission displayed the prominent clustering tendency. This phenomenon might be related with the prominent homosexual social cluster in these areas. In China, most MSM tend to move from their original place of residence, where their identities are easily recognized by acquaintances [39, 40], and concentrate in central cities, where close social and sexual network among MSM can be easily constructed [41]. A recent study applying scan statistics analysis revealed that the spatial clusters of reported MSM HIV/AIDS cases concentrated in municipalities, provincial capitals and main cities of China, such as Beijing, Shanghai, Chongqing, Chengdu, and Guangzhou [42]. Thus, intervention targeted to the transmission network should be promoted, such as active care, pre-exposure prophylaxis, post-exposure prophylaxis and early antiretroviral treatment.

For intravenous drug use, the only most likely cluster was detected in Dehong Prefecture, whose relative risk was 51.07, and far higher than those of the other clusters. Furthermore, this spatial cluster overlapped with the first secondary spatial cluster of recent infection contributed to heterosexual contact, which suggested that the transmission risks of heterosexual contact and intravenous drug use coexisted there. Historically, Dehong is considered as an entry of HIV-1 into China, and the Yunnan-Myanmar border area constitutes a hot spot of active HIV-1 recombination in Asia [43, 44]. In recent years, with the opening of labor markets in border areas, a certain amount of Burmese were introduced into China, which challenge the HIV/AIDS control and prevention in border areas [45, 46]. In this study, 87.8% (43/49) of foreign recently infected cases were found in four counties of Dehong Prefecture, which suggested that cross-border Burmese contributed most to the local HIV epidemics, and HIV/AIDS control and prevention in border areas should be further improved.

There are still some limitations to our study. First, as mentioned above, because of the lack for the exact number of persons tested over the study period, we could not directly estimate the HIV incidence, which is important for the evaluation of HIV epidemic. Second, the misclassification of recent infections cannot be completely avoided when using BED-CEIA [4, 47]. However, we took the associated exclusion criteria and combined recent infection assay with statistical analysis to draw a conclusion at the population level, which could reveal the factors associated with recent infections among the newly reported cases. Third, this study was based on the reported HIV cases. Because of various reasons, such as availability of services, social economic status, health awareness and stigma, there is still a gap between the reported HIV cases and the HIV population, which is also an obstacles to HIV/AIDS control and prevention around the world. We hope that our work could provide clues for further discovering HIV infected persons.

## Conclusion

In this study, the prevalence and demographic characteristic of recent HIV-1 infections among newly reported HIV cases was analyzed, which provided another way to understand the HIV epidemic in Yunnan. The factors associated with recent HIV infections included gender, age and transmission routes. The different screening approaches and contact histories were also associated with the proportion of recent HIV-1 infections among newly reported cases. For the first time, the spatial clusters of recent infections stratified by transmission routes were analyzed, which highlighted the areas with potential transmission risks. These findings provide information to develop strategies and measures to improve the efficiency of HIV testing and intervention.

## Additional files

Additional file 1: Spatial clusters of recent HIV-1 infections attributed to heterosexual contact by using the ellipse scanning window (PDF 408 kb)

Additional file 2: The spatial scan statistics analysis by using different percentages of the population at risk (PDF 70 kb)

## Data Availability

The datasets used and/or analysed during the current study available from the corresponding author on reasonable request.

## Abbreviations

BED-CEIA: BED-capture enzyme immunoassay
FSW: Female sexual worker
HIV: Human immunodeficiency virus
IDU: Intravenous drug user
MSM: Men who have sex with men
RR: Relative risk
