# Supplementary material for: Demographic characteristics and spatial clusters of recent HIV-1 infections among newly diagnosed HIV-1 cases in Yunnan, China, 2015
Source: BMC Public Health. 2019 Nov 11;19:1507. doi: 10.1186/s12889-019-7557-8 (PMC6849305; doi:10.1186/s12889-019-7557-8)
Supplement: Supplementary file 1 — Spatial clusters of recent HIV-1 infections attributed to heterosexual contact by using the ellipse scanning window (PDF 408 kb) [file 12889_2019_7557_MOESM1_ESM.pdf]

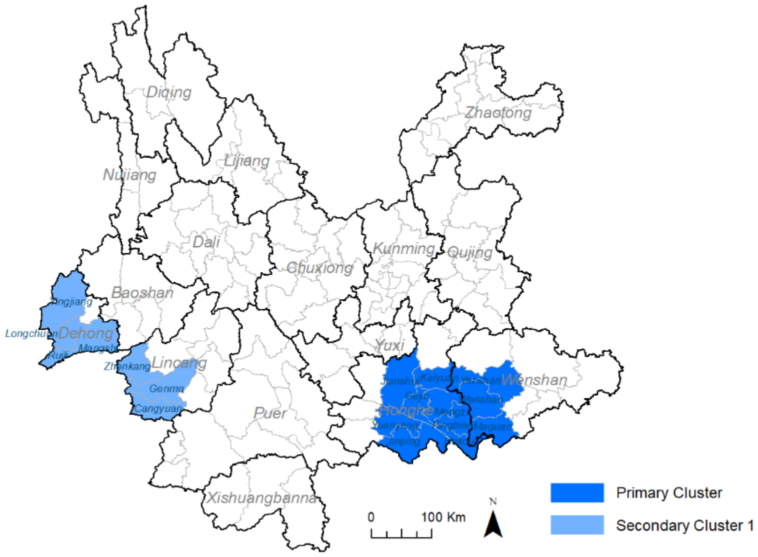

Additional file 1: Spatial clusters of recent HIV-1 infections attributed to heterosexual contact by using the ellipse scanning window. The shapefile of China was downloaded from the GADM database (www.gadm.org), version 3.4, April 2018, from which the shapefile of Yunnan was extracted with Quantum GIS.
