# Supplementary material for: Demographic characteristics and spatial clusters of recent HIV-1 infections among newly diagnosed HIV-1 cases in Yunnan, China, 2015
Source: BMC Public Health. 2019 Nov 11;19:1507. doi: 10.1186/s12889-019-7557-8 (PMC6849305; doi:10.1186/s12889-019-7557-8)
Supplement: Supplementary file 2 — The spatial scan statistics analysis by using different percentages of the population at risk (PDF 70 kb) [file 12889_2019_7557_MOESM2_ESM.pdf]

Additional file 2. The spatial scan statistics analysis by using different percentages of the population at risk.

Spatial Clusters		County Code	Number of cases	Relative Risk	Log Likelihood Ratio	*p*-value
**Heterosexual contact**						
5% of the population at risk	Primary Cluster	53252300, 53252200, 53253200, 53262100, 53262500	58	4.18	36.6	2.0E-15
	Secondary Cluster 1	53310200, 53312400, 53310300, 53312300	35	3.51	18.2	3.0E-07
	Secondary Cluster 2	53082700, 53082900, 53082800, 53282200, 53092700, 53092500, 53092600, 53080200	43	2.27	10.5	8.1E-04
10% of the population at risk	Primary Cluster	53252200, 53252300, 53250100, 53250200, 53262100, 53253200, 53252800, 53253000, 53262200, 53252400, 53262500	108	3.17	44.1	<1.0E-17
	Secondary Cluster 1	53310200, 53312400, 53310300, 53312300	35	3.51	18.2	6.5E-07
	Secondary Cluster 2	53082700, 53082900, 53082800, 53282200, 53092700, 53092500, 53092600, 53080200	43	2.27	10.5	2.0E-03

25% of the population at risk	Primary Cluster	53252200, 53252300, 53250100, 53250200, 53262100, 53253200, 53252800, 53253000, 53262200, 53252400, 53262500	108	3.17	44.1	<1.0E-17
	Secondary Cluster 1	53310200, 53312400, 53310300, 53312300	35	3.51	18.2	1.4E-06
	Secondary Cluster 2	53082700, 53082900, 53082800, 53282200, 53092700, 53092500, 53092600, 53080200	43	2.27	10.5	2.0E-03
50% of the population at risk	Primary Cluster	53252200, 53252300, 53250100, 53250200, 53262100, 53253200, 53252800, 53253000, 53262200, 53252400, 53262500	108	3.17	44.1	2.2E-16
	Secondary Cluster 1	53310200, 53312400, 53310300, 53312300	35	3.51	18.2	2.5E-06
	Secondary Cluster 2	53082700, 53082900, 53082800, 53282200, 53092700, 53092500, 53092600, 53080200	43	2.27	10.5	2.7E-03
**Heterosexual contact**						
5% of the population at risk	Primary Cluster	53018100, 53011200	18	20.77	34.0	6.9E-14

	Secondary Cluster 1	53290100	9	13.93	14.6	1.5E-05
10% of the population at risk	Primary Cluster	53018100, 53011200	18	20.77	34.0	3.3E-13
	Secondary Cluster 1	53290100	9	13.93	14.6	2.9E-05
25% of the population at risk	Primary Cluster	53018100, 53011200	18	20.77	34.0	7.7E-13
	Secondary Cluster 1	53290100	9	13.93	14.6	4.3E-05
50% of the population at risk	Primary Cluster	53018100, 53011200	18	20.77	34.0	2.6E-12
	Secondary Cluster 1	53290100	9	13.93	14.6	7.2E-05
**Intravenous drug injection**						
5% of the population at risk	Primary Cluster	53310200, 53312400, 53310300, 53312300	30	51.07	75.9	<1.0E-17
10% of the population at risk	Primary Cluster	53310200, 53312400, 53310300, 53312300	30	51.07	75.9	<1.0E-17
25% of the population at risk	Primary Cluster	53310200, 53312400, 53310300, 53312300	30	51.07	75.9	<1.0E-17
50% of the population at risk	Primary Cluster	53310200, 53312400, 53310300, 53312300	30	51.07	75.9	<1.0E-17
